# Long noncoding RNA Malat1 inhibits Tead3-Nfatc1–mediated osteoclastogenesis to suppress osteoporosis and bone metastasis

**DOI:** 10.21203/rs.3.rs-2405644/v1

**Published:** 2023-03-20

**Authors:** Yang Zhao, Hongqi Teng, Yalan Deng, Marisela Sheldon, Consuelo Martinez, Jie Zhang, Annie Tian, Yutong Sun, Shinichi Nakagawa, Fan Yao, Hai Wang, Li Ma

**Affiliations:** 1 Department of Experimental Radiation Oncology, The University of Texas MD Anderson Cancer Center, Houston, Texas 77030, USA; 2 Department of Kinesiology, Rice University, Houston, Texas 77005, USA; 3 Department of Molecular and Cellular Oncology, The University of Texas MD Anderson Cancer Center, Houston, Texas 77030, USA; 4 RNA Biology Laboratory, Faculty of Pharmaceutical Sciences, Hokkaido University, Sapporo 060-0812, Japan; 5 Department of Molecular and Cellular Biology, Roswell Park Comprehensive Cancer Center, Buffalo, New York 14263, USA; 6 The University of Texas MD Anderson Cancer Center UTHealth Houston Graduate School of Biomedical Sciences, Houston, Texas 77030, USA; 7 Present address: Hubei Hongshan Laboratory, College of Biomedicine and Health, Huazhong Agricultural University, Wuhan, Hubei 430070, China

## Abstract

MALAT1, one of the few highly conserved nuclear long noncoding RNAs (IncRNAs), is abundantly expressed in normal tissues. Previously, targeted inactivation and genetic rescue experiments identified MALAT1 as a suppressor of breast cancer lung metastasis. On the other hand, Malat1-knockout mice are viable and develop normally. On a quest to discover new roles of MALAT1 in physiological and pathological processes, we found that this lncRNA is downregulated during osteoclastogenesis in humans and mice. Notably, Malat1 deficiency in mice promotes osteoporosis and bone metastasis, which can be rescued by genetic add-back of *Malat1*. Mechanistically, Malat1 binds to Tead3 protein, a macrophage-osteoclast–specific Tead family member, blocking Tead3 from binding and activating Nfatc1, a master regulator of osteoclastogenesis, which results in the inhibition of Nfatc1-mediated gene transcription and osteoclast differentiation. Altogether, these findings identify Malat1 as a lncRNA that suppresses osteoporosis and bone metastasis.

## Introduction

Osteoporosis, characterized by decreased bone mineral density (BMD), increased bone fragility, and susceptibility to fracture, reflects an imbalance in which osteoclastic bone resorption exceeds osteoblastic bone formation^1, 2^ and is a potential contributor to acceleration of bone metastasis^3–5^. Primary osteoporosis occurs during the aging process, particularly in postmenopausal women^6^. Secondary osteoporosis has the same outcome as primary osteoporosis but is caused by certain medical conditions or medications^7^. In either condition, excessive osteoclastogenesis plays a key role and provides opportunities for therapeutic intervention.

Osteoclasts, a class of multinucleated giant cells (MGCs) that originate from the monocyte/macrophage lineage, are responsible for the resorption of bone matrix and minerals^8, 9^. Osteoclastogenesis is initiated by macrophage colony-stimulating factor (M-CSF) and receptor activator of nuclear factor-κB (RANK) ligand (RANKL), which induce the expression of osteoclast markers, such as cathepsin K (CTSK) and acid phosphatase 5 (ACP5, also known as TRAP), followed by maturation of osteoclast precursors and cell-cell fusion^10^. As a master regulator of osteoclastogenesis, nuclear factor of activated T cells 1 (NFATC1) is induced by RANKL, which in turn forms a complex with other transcription factors^11^ and activates the transcription of its own coding gene as well as other genes involved in osteoclast adhesion, cell fusion, and bone resorption^12–14^.

Long noncoding RNAs (lncRNAs), transcripts that are longer than 200 nucleotides and are not translated into proteins, function through binding to DNA, other RNA, and proteins^15, 16^. LncRNAs usually have low evolutionary conservation. One of the few exceptions, MALAT1, is a highly conserved nuclear lncRNA that is abundantly expressed in many tissues^17^. MALAT1 has been shown to modulate alternative pre-mRNA splicing based on siRNA knockdown results from cultured cell lines^18^. In 2012, three groups reported that Malat1-knockout mice showed no obvious phenotypic differences compared with wild-type mice under physiological conditions, and loss of Malat1 in mice did not affect alternative pre-mRNA splicing^19–21^. On the other hand, recent animal studies suggested that Malat1 has important functions under pathological conditions. For instance, through targeted inactivation, restoration (genetic rescue), and overexpression of Malat1 in mouse models, we found that Malat1 suppresses breast cancer lung metastasis through binding and inactivation of the Tead family of transcription factors^22^. Moreover, Malat1-null mice exhibited enhanced antiviral responses, suggesting that Malat1 may suppress antiviral innate immunity^23^. In addition, when fed a high-fat diet, *Apoe*^−/−^ mice transplanted with *Apoe*^−/−^;*Malat1*^−/−^ bone marrow showed higher atherosclerotic plaque burden in the aorta and increased hematopoietic progenitor cells and their progeny, suggesting that Malat1 may regulate hematopoietic cells^24^.

Recent genome-wide association studies (GWAS) showed that single nucleotide polymorphisms (SNPs) are associated with osteoporosis^1, 25^. Interestingly, one such analysis identified an SNP (rs202070768) at the *MALAT1* locus that was associated with low BMD^26^. However, functional evidence of MALAT1 alterations having a role in low BMD and osteoporosis is lacking. In the present study, by using genetically engineered mouse models, we identified Malat1 as a negative regulator of osteoporosis and bone metastasis. Mechanistically, Malat1 binds and sequesters Tead3, blocking Tead3 from interacting with and activating Nfatc1. Consequently, loss of Malat1 derepresses Tead3, which in turn promotes Nfatc1-mediated osteoclast differentiation. Importantly, the osteoporosis and bone metastasis phenotypes of Malat1-deficient mice can be rescued by genetic add-back of *Malat1*. Taken together, our findings reveal that Malat1 inhibits Tead3-Nfatc1–mediated osteoclastogenesis to suppress osteoporosis and bone metastasis.

## Results

### MALAT1 is downregulated during osteoclast differentiation in humans and mice

**Extended Data Figure 1.**
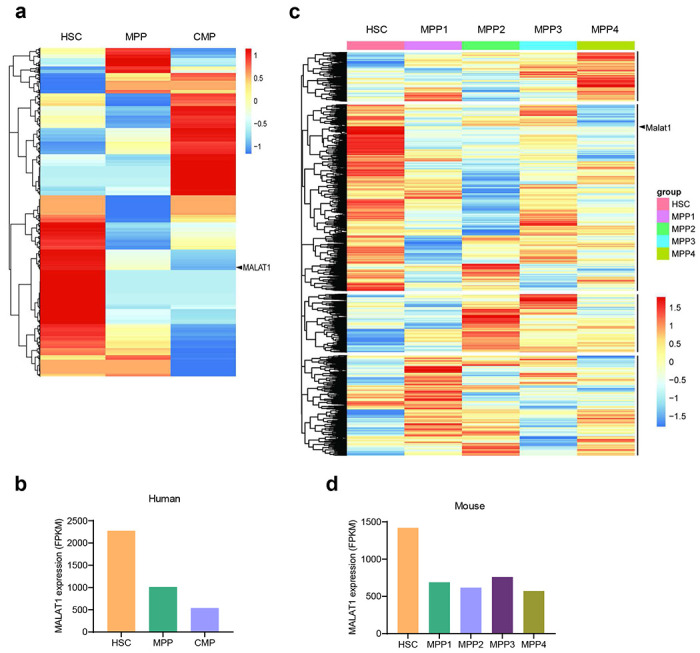
Malat1 is downregulated in hematopoietic progenitor cells compared with hematopoietic stem cells in humans and mice. **a**. Heatmap of differentially expressed genes among human hematopoietic stem cells (HSC), multipotent progenitors (MPP), and common myeloid progenitors (CMP). Data source: Expression Atlas dataset E-MTAB-3819. **b**. Relative expression levels of MALAT1 were quantitated from the RNA-seq results in **a**. **c**. Heatmap of differentially expressed genes among mouse hematopoietic stem cells (HSC) and four groups of multipotent progenitors (MPP). Data source: Expression Atlas dataset E-MTAB-7391. **d**. Relative expression levels of MALAT1 were quantitated from the RNA-seq results in **c**.

Hematopoietic stem cells (HSCs) undergo self-renewal and differentiation in the bone marrow. During a hierarchical differentiation process, HSCs turn into multipotent progenitors (MPPs), which then differentiate into oligopotent progenitors, including common myeloid progenitors (CMPs) and common lymphoid progenitors (CLPs)^27^. Recent reports of Malat1 having a role in regulating hematopoietic cells under pathological conditions^23, 24^ prompted us to analyze MALAT1 expression during differentiation of HSCs by using publicly available high-throughput sequencing datasets. Interestingly, in both humans and mice, MALAT1 was expressed at higher levels in HSCs than in MPPs or CMPs (Extended Data Fig. 1a–d).

**Figure 1.**
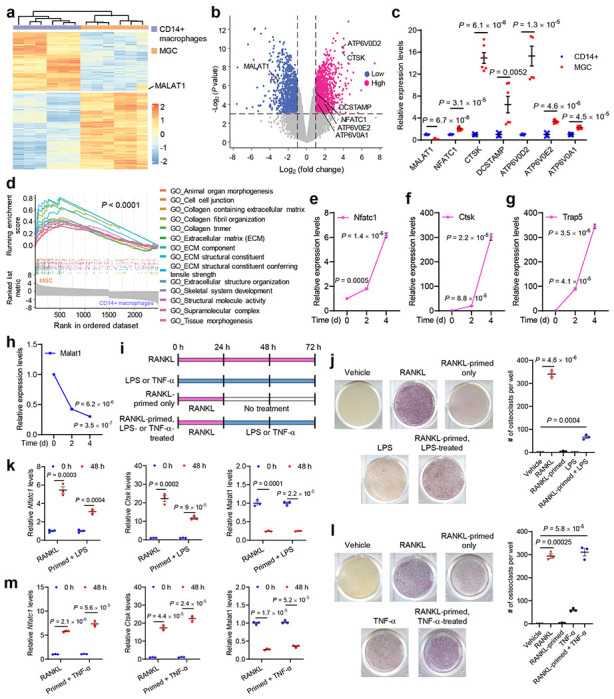
MALAT1 is downregulated during osteoclast differentiation, **a-d.** CD14^+^ human placental macrophages were differentiated into multinucleated giant cells (MGCs) in culture. Both CD14^+^ macrophages and MGCs were subjected to high-throughput RNA sequencing (RNA-seq). *n* = 6 biological replicates per group. Data source: GSE38747. **a.** Heatmap of differentially expressed genes between CD14^+^ macrophages and MGCs. **b.** Volcano plot of genes upregulated (red) or downregulated (blue) in MGCs relative to CD14^+^ macrophages. Cut-off values: |log_2_ (fold change)| > 1 and *P* value < 0.001. **c.** Relative expression levels of MALAT1 and osteoclast markers were quantitated from the RNA-seq results. **d.** Gene set enrichment analysis (GSEA) of the RNA-seq data, showing the top 14 Gene Ontology (GO) pathways of 486 significant pathways. **e-h.** qPCR of *Nfatc1* (**e**), *Ctsk* (**f**), *Trap5* (**g**), and Malat1 (**h**) in RAW264.7 cells treated with soluble RANKL (50 ng/mL) for the indicated times. **i.** Schematic representation of the treatments used to evaluate the osteoclastogenic activity of RANKL, LPS, and TNF-α, with or without pretreatment (priming) with RANKL. **j.** TRAP staining images (left panel) and quantification (right panel) of RAW264.7 cells treated with RANKL or LPS, with or without pretreatment with RANKL. **k.** qPCR of *Nfatc1*, *Ctsk*, and Malat1in the cells described in **j**. **l.** TRAP staining images (left panel) and quantification (right panel) of RAW264.7 cells treated with RANKL or TNF-α, with or without pretreatment with RANKL. **m.** qPCR of *Nfatc1*, *Ctsk*, and Malat1in the cells described in **l**. Statistical significance in **c** and **j-m** was determined by a two-tailed unpaired *t*-test. Error bars are s.e.m.

CMPs can differentiate into monocytes and macrophages, which are the precursors of osteoclasts^28^. We analyzed gene expression during the differentiation of human placental CD14^+^ macrophages into multinucleated giant cells (MGCs)^29^ (Fig. 1a), in which osteoclasts are the major cell population^8^. Compared with CD14^+^ macrophages, MGCs showed elevated expression of osteoclast markers, including *NFATC1, CTSK, DCSTAMP*, *ATP6V0D2, ATP6V0E2,* and *ATP6V0A1* (Fig. 1b, c). In contrast, MALAT1 was significantly downregulated in MGCs relative to CD14^+^ macrophages (Fig. 1a–c). Consistent with the functions of osteoclasts, gene set enrichment analysis (GSEA) indicated that genes upregulated in MGCs compared with CD14^+^ macrophages were related to collagen organization, extracellular structure remodeling, and skeletal development (Fig. 1d and **Supplementary Table 1**). To further validate the downregulation of Malat1 during the differentiation of macrophages into osteoclasts, we treated a mouse macrophage/pre-osteoclast cell line, RAW264.7, with soluble RANKL to induce osteoclast differentiation^30^. After this treatment, markers of osteoclasts, including *Nfatc1, Ctsk*, and *Trap5*, were upregulated in a time-dependent manner (Fig. 1e–g), whereas Malat1 expression levels were markedly decreased (Fig. 1h). Taken together, these results reveal MALAT1 as a lncRNA that is downregulated during osteoclastogenesis in humans and mice.

Lipopolysaccharides (LPS) and TNF-α are involved in pathological osteoclastogenesis^30–33^. Consistent with previous reports^31, 32^, we observed that LPS treatment alone was insufficient to initiate osteoclast differentiation; instead, when RAW264.7 cells were pretreated with RANKL, LPS treatment promoted osteoclastogenesis, as gauged by staining for tartrate-resistant acid phosphatase (TRAP), a widely used marker of osteoclasts (Fig. 1i, j), and upregulated the expression of *Nfatc1* and *Ctsk* (Fig. 1k). TNF-α, on the other hand, can induce osteoclast differentiation in both RANKL-dependent and RANKL-independent manners^34, 35^. Indeed, we found that treating RAW264.7 cells with TNF-α induced osteoclastogenesis and the expression of *Nfatc1* and *Ctsk*, either with or without RANKL pretreatment (Fig. 1i, l, m). Notably, Malat1 was substantially downregulated during LPS- or TNF-α-induced osteoclast differentiation (Fig. 1k, m). These findings suggest that Malat1 may be involved in osteoclastogenesis in response to various stimuli.

### Genetic models reveal that Malat1 protects against osteoporosis and bone metastasis

To study the role of Malat1 in osteoclastogenesis and osteoporosis *in vivo*, we used a Malat1-knockout mouse model (*Malat1*^−/−^) described in our previous study, in which a transcriptional terminator was inserted downstream of the transcriptional start site of *Malat1*, causing the loss of Malat1 RNA expression without altering expression levels of Malat1’s adjacent genes^22^. Also, we previously engineered mice with targeted transgenic *Malat1* expression from the *ROSA26* locus (*Malat1*^Tg/Tg^), which enabled us to conduct genetic rescue studies in *Malat1*^−/−^ mice by generating *Malat1*^−/−^;*Malat1*^Tg/Tg^ animals^22^.

**Extended Data Figure 2.**
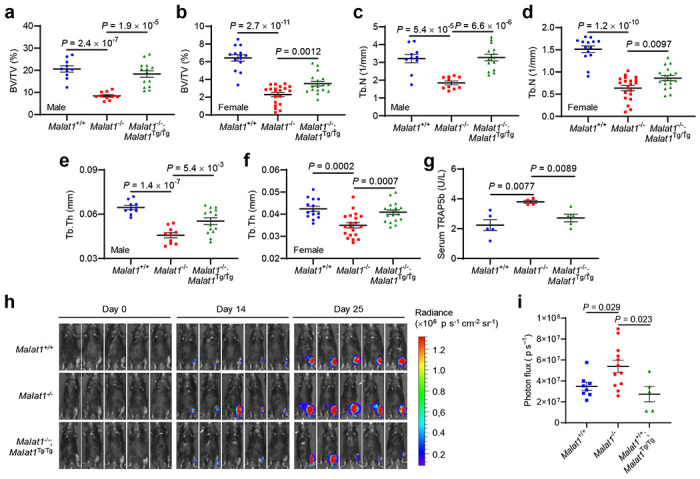
Malat1 is a negative regulator of osteoporosis and bone metastasis. **a-f.** μCT-based measurements of the trabecular bone volume per tissue volume (BV/TV, **a** and **b**), trabecular number (Tb.N, **c** and **d**), and trabecular thickness (Tb.th, **e** and **f**) in the femurs from 6-month-old male (*n* = 5-7 mice per group) and female (*n* = 7-10 mice per group) *Malat1*^+/+^, *Malat1*^−/−^, and *Malat1*^−/−^;*Malat1*^Tg/Tg^ mice, with left and right femurs for each mouse measured. **g.** ELISA of serum TRAP5b levels in 6-month-old male *Malat1*^+/+^, *Malat1*^−/−^, and *Malat1*^−/−^;*Malat1*^Tg/Tg^ mice. *n* = 4-5 mice per group. **h, i.** 6-month-old *Malat1*^+/+^, *Malat1*^−/−^, and *Malat1*^−/−^;*Malat1*^Tg/Tg^ mice received intratibial injection of 5,000 B16F1 melanoma cells. Bioluminescent imaging of live animals was performed at the indicated times (**h**) and photo flux was quantitated at day 25 (**i**). Statistical significance in **a-g** and **i** was determined by a two-tailed unpaired *t*-test. Error bars are s.e.m.

**Figure 2.**
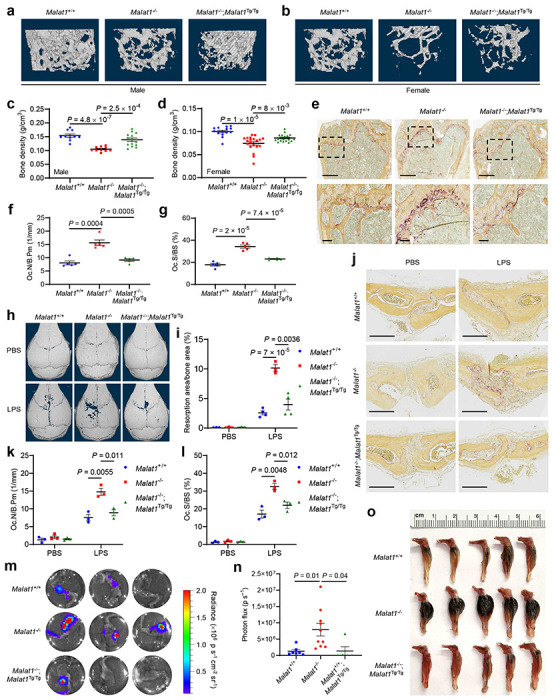
Targeted inactivation and restoration of Malat1 reveal that Malat1 protects against osteoporosis and bone metastasis. **a, b.** Representative μCT images of 3D bone structures of the femurs from 6-month-old male (**a**) and female (**b**) *Malat1*^+/+^, *Malat1*^−/−^, and *Malat1*^−/−^;*Malat1*^Tg/Tg^ mice. **c, d.** μCT-based measurements of the bone mineral density of the femurs from 6-month-old male (**c**; *n* = 5-7 mice per group) and female (**d**; *n* = 7-10 mice per group) *Malat1*^+/+^, *Malat1*^−/−^, and *Malat1*^−/−^;*Malat1*^Tg/Tg^ mice, with left and right femurs for each mouse measured. **e.** Representative TRAP staining images of the femurs from 6-month-old male *Malat1*^+/+^, *Malat1*^−/−^, and *Malat1*^−/−^;*Malat1*^Tg/Tg^ mice. Scale bars, 700 μm in upper panels and 100 μm in lower panels. **f, g.** Quantification of osteoclast numbers per bone perimeter (Oc.N/B.Pm, **f**) and osteoclast surface per bone surface (Oc.S/BS, **g**) in femurs of the mice described in **e**. *n* = 5 mice per group. **h-l.** μCT images of the surface of calvariae (**h**), quantification of the relative resorption area (**i**), TRAP staining images of calvarial sections (**j**), the number of osteoclasts per bone perimeter (Oc.N/B.Pm, **k**), and osteoclast surface per bone surface (Oc.S/BS, **l**) in the calvarial bones from 8-week-old female *Malat1*^+/+^, *Malat1*^−/−^, and *Malat1*^−/−^;*Malat1*^Tg/Tg^ mice after the administration of PBS or LPS to the calvarial periosteum for 5 days, *n* = 3-4 mice per group. Scale bars in **j**, 200 μm. **m-o.** Bioluminescent imaging (**m**), quantification of photon flux (**n**), and photos (**o**) of the tibiae of 6-month-old *Malat1*^+/+^, *Malat1*^−/−^, and *Malat1*^−/−^;*Malat1*^Tg/Tg^ mice at day 26 after intratibial injection of 5,000 B16F1 melanoma cells. Statistical significance in **c**, **d**, **f**, **g**, **i**, **k**, **l**, and **n** was determined by a two-tailed unpaired *t*-test. Error bars are s.e.m.

By performing microcomputed tomographic (μCT) analysis of the femurs of 6-month-old animals, we found that both male and female *Malat1*^−/−^;*Malat1*^Tg/Tg^ mice had much less bone mass than age- and sex-matched *Malat1*^+/+^ mice; importantly, this osteoporotic phenotype was rescued in *Malat1*^−/−^;*Malat1*^Tg/Tg^ mice (Fig. 2a, b). Quantification of the μCT data revealed that compared with *Malat1*^+/+^ mice, the trabecular bone density (Fig. 2c, d), trabecular bone volume per tissue volume (BV/TV, Extended Data Fig. 2a, b), trabecular number (Tb.N, Extended Data Fig. 2c, d), and trabecular thickness (Tb.th, Extended Data Fig. 2e, f) were significantly reduced in *Malat1*^−/−^ mice, and these reductions were largely reversed by genetic restoration of Malat1 expression (Fig. 2c, d and Extended Data Fig. 2a–f). Staining for TRAP revealed a significant increase in osteoclasts in the femurs of *Malat1*^−/−^ mice compared with *Malat1*^+/+^ mice, and this increase was reversed in *Malat1*^−/−^;*Malat1*^Tg/Tg^ mice (Fig. 2e). Quantification of femoral osteoclasts showed that relative to *Malat1*^+/+^ mice, the osteoclast numbers per bone perimeter (Oc.N/B.Pm, Fig. 2f) and the osteoclast surface per bone surface (Oc.S/BS, Fig. 2g) were elevated in *Malat1*^−/−^ mice by approximately 2-fold. Moreover, enzyme-linked immunosorbent assay (ELISA) revealed that *Malat1*^−/−^ mice had higher levels of the serum bone resorption marker TRAP5b than *Malat1*^+/+^ and *Malat1*^−/−^;*Malat1*^Tg/Tg^ mice (Extended Data Fig. 2g).

Next, to determine the role of Malat1 in modulating pathological bone loss, we used a well-established mouse model of inflammatory bone resorption, which involves the injection of lipopolysaccharides (LPS) into the subcutaneous space over the calvarial bones^36^. As gauged by μCT imaging, administration of LPS to 8-week-old *Malat1*^−/−^ mice resulted in significantly aggravated erosions on the surface of the calvarial bones, compared with *Malat1*^+/+^ or *Malat1*^−/−^;*Malat1*^Tg/Tg^ mice (Fig. 2h, i). TRAP staining and quantification revealed that after LPS injection, *Malat1*^−/−^ mice had higher osteoclast numbers per bone perimeter (Oc.N/B.Pm, Fig. 2j, k) and more osteoclast surface per bone surface (Oc.S/BS, Fig. 2j, l) than either *Malat1*^+/+^ or *Malat1*^−/−^;*Malat1*^Tg/Tg^ mice. Collectively, these findings indicate that Malat1 deficiency promotes osteoporosis under both physiological and inflammatory conditions.

Untreated osteoporosis is associated with accelerated progression of bone metastasis in cancer patients^3–5^. Drugs for osteoporosis therapy, such as bisphosphonates that inhibit osteoclast-mediated bone resorption, have been used to treat bone diseases including bone metastases^37^. To determine whether Malat1 in the host confers protection from bone metastases, we injected luciferase-labeled B16F1 melanoma cells into the tibiae of 6-month-old *Malat1*^+/+^, *Malat1*^−/−^, or *Malat1*^−/−^;*Malat1*^Tg/Tg^ mice, and we found that bone metastases were markedly exacerbated by Malat1 loss in the hosts, a phenotype that was rescued by Malat1 re-expression, as gauged by bioluminescent imaging of live animals (Extended Data Fig. 2h, i) and dissected bones (Fig. 2m, n), as well as gross examination of visible bone lesions (Fig. 2o). These results indicate that Malat1 has a non-tumor cell-autonomous role in bone metastasis suppression.

**Extended Data Figure 3.**
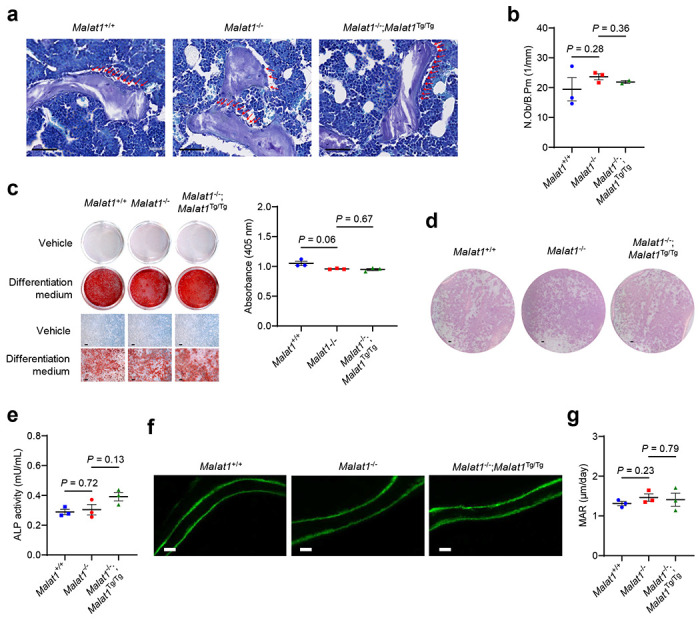
Malat1 is not involved in osteoblastic bone formation. **a**. Toluidine blue staining of sections of the femurs from 2-month-old male *Malat1*^+/+^, *Malat1*^−/−^ and *Malat1*^−/−^;*Malat1*^Tg/Tg^ mice. Scale bars, 50 μm. Red arrows indicate osteoblasts. **b**. Quantification of the osteoblast numbers per bone perimeter (N.Ob/B.Pm) in the mice described in **a**. **c**. Mesenchymal stem cells (MSCs) were isolated from *Malat1*^+/+^, *Malat1*^−/−^, and *Malat1*^−/−^;*Malat1*^Tg/Tg^ mice, cultured in osteogenic differentiation medium for 14 days, and subjected to alizarin red S (ARS) staining (left panel). Scale bars, 100 μm. ARS stains were dissolved and quantitated as absorbance at 405 nm (right panel). **d**. Mesenchymal stem cells (MSCs) were isolated from *Malat1*^+/+^, *Malat1*^−/−^, and *Malat1*^−/−^;*Malat1*^Tg/Tg^ mice, cultured in differentiation medium for 14 days, and subjected to alkaline phosphatase (ALP) staining. Scale bars, 1 mm. **e**. Activity of secreted ALP was measured in the culture medium collected after the MSC differentiation described in **d**. **f**. Representative images of bone formation rates of 2-month-old male *Malat1*^+/+^, *Malat1*^−/−^, and *Malat1*^−/−^;*Malat1*^Tg/Tg^ mice, as determined by sequential labeling with calcein. Scale bars, 10 μm. **g**. Bone histomorphometric analysis of mineral apposition rate (MAR) in the femurs of 2-month-old male *Malat1*^+/+^, *Malat1*^−/−^, and *Malat1*^−/−^;*Malat1*^Tg/Tg^ mice. *n* = 3 mice per group. Statistical significance in **b**, **c**, **e**, and **g** was determined by a two-tailed unpaired **t**-test. Error bars are s.e.m.

Because bone homeostasis is maintained by osteoclastic bone resorption and osteoblastic bone formation, we next determined whether Malat1 modulates the number and differentiation potential of osteoblasts. To this end, we stained bone sections with toluidine blue^38^ (Extended Data Fig. 3a), which revealed no significant difference in the numbers of osteoblasts per bone perimeter (N.Ob/B.Pm) among *Malat1*^+/+^, *Malat1*^−/−^, and *Malat1*^−/−^;*Malat1*^Tg/Tg^ mice (Extended Data Fig. 3a, b). Further, we isolated mouse mesenchymal stem cells (MSCs) from these three mouse lines and cultured them in osteogenic differentiation medium^39^; we observed comparable osteogenic differentiation in all three groups, as gauged by calcium mineralization (via alizarin red staining, Extended Data Fig. 3c) and alkaline phosphatase (ALP, Extended Data Fig. 3d, e). Moreover, we found no significant difference in bone formation rates among *Malat1*^+/+^, *Malat1*^−/−^, and *Malat1*^−/−^;*Malat1*^Tg/Tg^ mice, as gauged by dynamic histomorphometry measurements through sequential labeling with calcein, a fluorescent chromophore that binds to calcified skeletal structures^40, 41^ (Extended Data Fig. 3f, g). Taken together, our results suggest that Malat1 inhibits osteoclast differentiation and protects against osteoporosis and bone metastasis without affecting osteoblastic bone formation.

### Malat1 deficiency promotes osteoclastogenesis through the activation of Nfatc1

**Figure 3.**
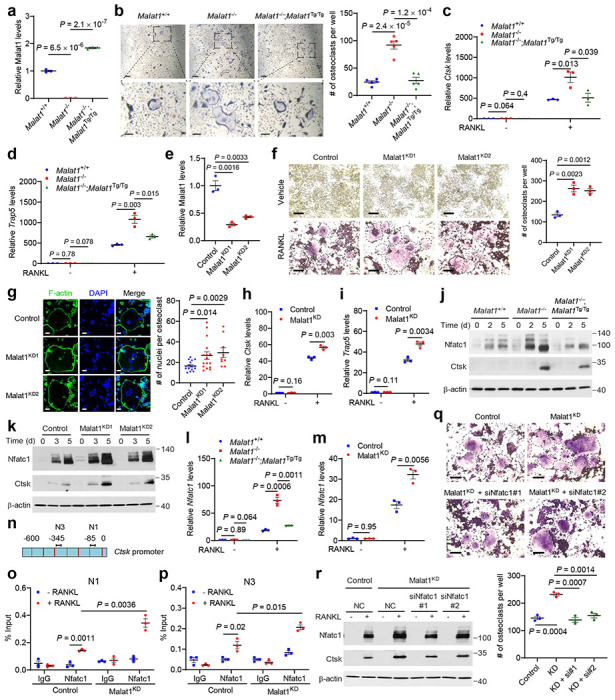
Malat1 deficiency promotes osteoclastogenesis through the activation of Nfatc1. **a.** qPCR of Malat1 in primary BMMs isolated from *Malat1*^+/+^, *Malat1*^−/−^, and *Malat1*^−/−^;*Malat1*^Tg/Tg^ mice. **b.** TRAP staining images (left panel) and quantification (right panel) of *Malat1*^+/+^, *Malat1*^−/−^, and *Malat1*^−/−^;*Malat1*^Tg/Tg^ BMMs treated with M-CSF (50 ng/mL) and RANKL (100 ng/mL) for 4 days. Multinucleated TRAP-positive cells were counted. Scale bars, 125 μm in upper panels and 50 μm in lower panels. **c, d.** qPCR of *Ctsk* (**c**) and *Trap5* (**d**) in the BMMs described in **b**. **e.** qPCR of Malat1 in control and Malat1-knockdown RAW264.7 cells generated by CRISPR interference. **f.** TRAP staining images (left panel) and quantification (right panel) of control and Malat1-knockdown RAW264.7 cells treated with RANKL (50 ng/mL) for 4 days. Multinucleated TRAP-positive cells (outlined by dashed lines) were counted. Scale bars, 100 μm. **g.** Left panel: after stimulation with RANKL (50 ng/mL) for 4 days, control and Malat1-knockdown RAW264.7 cells were stained with Phalloidin Green 488 (indicating F-actin rings) and DAPI (blue; indicating nuclei). Right panel: quantification of the number of nuclei per osteoclast. Scale bar, 50 μm. **h, i.** qPCR of *Ctsk* (**h**) and *Trap5* (**i**) in control and Malat1-knockdown RAW264.7 cells treated with RANKL (50 ng/mL) for 3 days. **j, k.** Immunoblotting of Nfatc1, Ctsk, and β-actin in *Malat1*^+/+^, *Malat1*^−/−^, and *Malat1*^−/−^;*Malat1*^Tg/Tg^ BMMs treated with M-CSF (50 ng/mL) and RANKL (100 ng/mL) for 2 days and 5 days (**j**), and in control and Malat1-knockdown RAW264.7 cells cultured with RANKL (50 ng/mL) for 3 and 5 days (**k**). **l, m.** qPCR of *Nfatc1* in *Malat1*^+/+^, *Malat1*^−/−^, and *Malat1*^−/−^;*Malat1*^Tg/Tg^ BMMs treated with M-CSF (50 ng/mL) and RANKL (100 ng/mL) for 4 days (**l**), and in control and Malat1-knockdown RAW264.7 cells treated with RANKL (50 ng/mL) for 3 days (**m**). **n.** Graphical representation of the mouse *Ctsk* promoter. Primers previously reported for amplifying N1 and N3 regions were used for ChIP-qPCR. **o, p.** ChIP-qPCR analysis showing the occupancy of the N1 (**o**) and N3 (**p**) regions of the *Ctsk* gene promoter by Nfatc1 immunoprecipitated from control or Malat1-knockdown RAW264.7 cells treated with RANKL (50 ng/mL) for 3 days. **q, r.** Control and Malat1-knockdown RAW264.7 cells were transfected with Nfatc1 siRNA or scrambled negative control (NC). At 24 hours after siRNA transfection, the cells were treated with RANKL for 5 days, followed by TRAP staining and quantification (**q**) of multinucleated TRAP-positive cells (outlined by dashed lines). Scale bars, 100 μm. Cell lysates were subjected to immunoblotting of Nfatc1, Ctsk, and β-actin (**r**). Statistical significance in **a-i**, **l**, **m**, and **o-q** was determined by a two-tailed unpaired *t*-test. Error bars are s.e.m.

Because the *Malat1*^−/−^ and *Malat1*^Tg/Tg^ animals used in our study are whole-body knockout and transgenic mice, the osteoporotic phenotype observed above may or may not be a direct effect of Malat1 loss in osteoclast precursors. To address this issue, we isolated primary bone marrow-derived macrophages (BMMs) from *Malat1*^+/+^, *Malat1*^−/−^, and *Malat1*^−/−^;*Malat1*^Tg/Tg^ mice, and then treated these osteoclast precursors with M-CSF and RANKL for 4-6 days to induce their differentiation into osteoclasts. Genetic ablation and restoration of Malat1 expression in BMMs were confirmed by qPCR (Fig. 3a). After M-CSF- and RANKL-induced differentiation, we detected osteoclasts by TRAP staining (Fig. 3b), finding that knockout of Malat1 led to a prominent increase in the number of TRAP-positive multinucleated osteoclasts, and that reexpression of Malat1 reversed the observed induction of osteoclastogenesis (Fig. 3b). The mRNA levels of osteoclast markers *Ctsk* and *Trap5* were much higher in *Malat1*^−/−^ cells than in *Malat1*^+/+^ and *Malat1*^−/−^;*Malat1*^Tg/Tg^ cells after RANKL treatment (Fig. 3c, d).

**Extended Data Figure 4.**
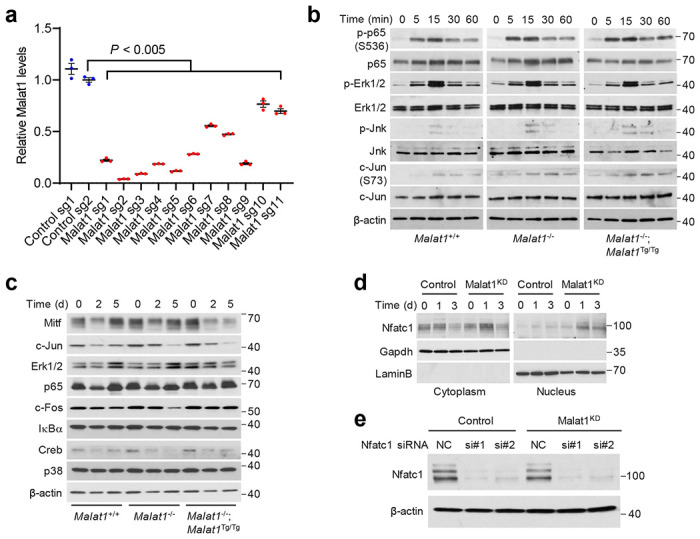
Effects of CRISPRi-mediated knockdown of Malat1 on proosteoclastogenic signaling and Nfatc1. **a**. qPCR of Malat1 in B16F1 cells with CRISPRi-mediated knockdown of Malat1. Statistical significance was determined by a two-tailed unpaired *t*-test. Error bars are s.e.m. **b**. *Malat1*^+/+^, *Malat1*^−/−^, and *Malat1*^−/−^;*Malat1*^Tg/Tg^ BMMs were cultured with M-CSF (50 ng/mL) before stimulation with RANKL (100 ng/mL) for 0, 5, 15, 30, and 60 min. The cell lysates were subjected to immunoblotting with antibodies against phospho-p65, p65, phospho-Erk1/2, Erk1/2, phospho-Jnk, Jnk, phospho-c-Jun, c-Jun, and β-actin. **c**. *Malat1*^+/+^, *Malat1*^−/−^, and *Malat1*^−/−^;*Malat1*^Tg/Tg^ BMMs were treated with M-CSF (50 ng/mL) and RANKL (100 ng/mL) for 2 days and 5 days. The cell lysates were subjected to immunoblotting with antibodies against Mitf, c-Jun, Erk1/2, p65, c-Fos, IκBα, Creb, p38, and β-actin. **d**. Control and Malat1-knockdown RAW264.7 cells were cultured with RANKL (50 ng/mL) for 1 day and 3 days. After fractionation, the cytoplasmic and nuclear fractions were subjected to immunoblotting with antibodies against Nfatc1, Gapdh (a cytoplasmic marker), and Lamin B (a nuclear marker). **e**. Immunoblotting of Nfatc1 and β-actin in control and Malat1-knockdown RAW264.7 cells transfected with Nfatc1 siRNA or scrambled negative control (NC).

We also used CRISPR interference (CRISPRi) to knock down Malat1 in cell lines. Eleven single guide RNAs (sgRNAs) targeting mouse *Malat1* were tested by using the mouse B16F1 cell line (Extended Data Fig. 4a). sgRNA-2 and sgRNA-3 were chosen to knock down Malat1 in RAW264.7 cells, which was validated by qPCR (Fig. 3e), and the two resulting Malat1-knockdown stable cell lines were named Malat1^KD1^ and Malat1^KD2^. After RANKL-induced differentiation, both Malat1^KD1^ and Malat1^KD2^ cells gave rise to more TRAP-positive multinucleated osteoclasts than the control RAW264.7 cells (Fig. 3f). Fluorescent staining of F-actin rings (microfilament structures that are characteristic of mature osteoclasts^42, 43^) and nuclei, by phalloidin and DAPI, respectively, revealed that Malat1^KD1^ and Malat1^KD2^ cells had higher numbers of nuclei per osteoclast than the control cells (Fig. 3g). Moreover, *Ctsk* and *Trap5* mRNA levels were upregulated by knockdown of Malat1 in RANKL-treated RAW264.7 cells (Fig. 3h, i). Collectively, the results from primary BMMs and RAW264.7 cells suggest that Malat1 deficiency in osteoclast precursors promotes RANKL-induced osteoclastogenesis.

Upon binding to the RANK receptor, RANKL stimulates multiple signaling cascades, including nuclear factor-κB (NF-κB) signaling, mitogen-activated protein kinases (MAPK) signaling, and activator protein-1 (AP-1, whose major components are c-Jun and c-Fos proteins) signaling, leading to activation of downstream transcription factors, such as Nfatc1, Mitf, and Creb^14^. To understand how Malat1 inhibits osteoclastogenesis, we first stimulated BMMs with RANKL for short periods (5-60 minutes) and examined the phosphorylation events in the signaling pathways mentioned above, finding no substantial difference in the phosphorylation levels of p65 (also known as RelA), Erk1/2, Jnk, or c-Jun among the BMMs isolated from *Malat1*^+/+^, *Malat1*^−/−^, and *Malat1*^−/−^;*Malat1*^Tg/Tg^ mice (Extended Data Fig. 4b). Thus, Malat1 loss does not affect the early kinase signaling events during RANKL-induced osteoclast differentiation.

Next, we extended the RANKL stimulation time to 2 days and 5 days, finding that Malat1 deficiency did not affect the levels of Mitf, Erk1/2, c-Fos, IκBα, Creb, and p38 (Extended Data Fig. 4c). On the other hand, compared with *Malat1*^+/+^ and *Malat1*^−/−^;*Malat1*^Tg/Tg^ BMMs, Malat1-knockout BMMs showed more induction of Nfatc1 and its transcriptional target Ctsk^44^ (Fig. 3j). We observed similar results from RANKL-treated Malat1^KD1^ and Malat1^KD2^ RAW264.7 cells (Fig. 3k), and these cells exhibited nuclear accumulation of Nfatc1 (Extended Data Fig. 4d). Nfatc1 is known to act as its own transcription factor^11, 45^. Consistently, Malat1-knockout BMMs and Malat1-knockdown RAW264.7 cells showed more induction of *Nfatc1* mRNA levels after RANKL stimulation, compared with their Malat1 wild-type counterparts (Fig. 3l, m). *Ctsk*, a classic Nfatc1 target gene, contains two Nfatc1-binding sites in the promoter region^46^ (Fig. 3n). Chromatin immunoprecipitation-qPCR assays revealed that after RANKL treatment, Malat1-knockdown RAW264.7 cells showed more occupancy of these two regions by Nfatc1 than the control RAW264.7 cells (Fig. 3o, p). Importantly, knockdown of Nfatc1 in Malat1-depleted RAW264.7 cells reversed the induction of osteoclastogenesis and Ctsk expression upon stimulation with RANKL (Fig. 3q, r and Extended Data Fig. 4e). Taken together, these results suggest that Malat1 loss promotes osteoclast differentiation through Nfatc1.

### Malat1 binds to Tead3 to inhibit Nfatc1 activity and osteoclastogenesis

**Extended Data Figure 5.**
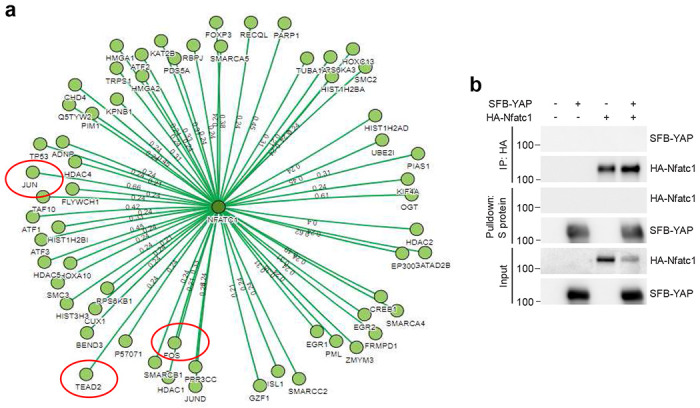
Analysis of the interaction of NFATC1 with other proteins. **a**. Schematic representation of NFATC1-binding proteins, based on the Mentha database (http://mentha.uniroma2.it/index.php). The threshold value was an evidence score of > 0.2. **b**. HEK293T cells were co-transfected with HA-Nfatc1 and SFB-YAP and subjected to pulldown with an HA-specific antibody or S-protein beads, followed by immunoblotting with antibodies against FLAG and HA.

How does Malat1 regulate Nfatc1? The binding of Nfatc1 to other proteins can lead to either activation or inhibition of the transcriptional activity of Nfatc1^47^, while lncRNAs often exert their functions by interacting with proteins, and this mode of action has been demonstrated for Malat1^15, 17, 22, 48^. We speculated that Malat1 might regulate the Nfatc1 auto-amplification loop by interacting with Nfatc1 and/or its binding proteins, and thus we searched a database of protein-protein interactions, Mentha (http://mentha.uniroma2.it/index.php). Of all potential NFATC1-interacting proteins (Extended Data Fig. 5a), TEAD was of particular interest, because our previous chromatin isolation by RNA purification coupled to mass spectrometry (ChIRP-MS) analysis captured an endogenous Malat1-Tead interaction in mouse tissues, which was validated by ChIRP-Western, RNA pulldown, and RNA immunoprecipitation assays^22^. Hence, we hypothesized that Malat1 might regulate Nfatc1 through Tead.

**Figure 4.**
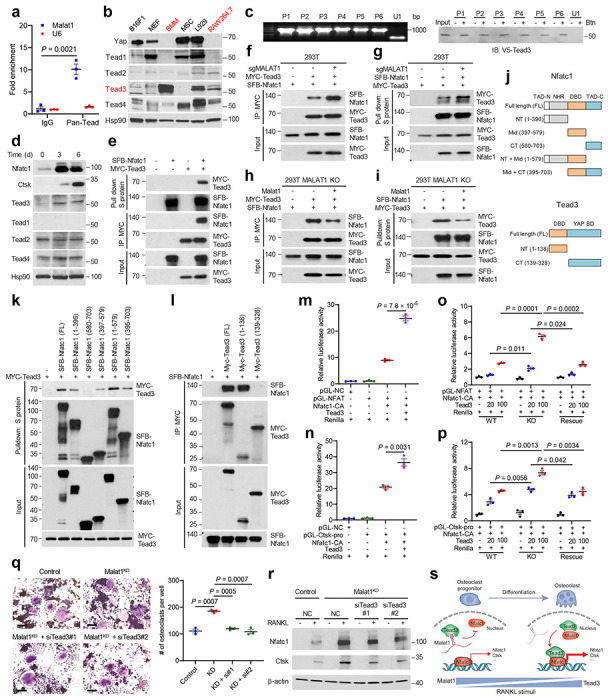
Malat1 binds to Tead3 to inhibit Nfatc1 activity and osteoclastogenesis. **a.** RNA immunoprecipitation (RIP) assay. Tead proteins were immunoprecipitated from cross-linked RAW264.7 cells by a pan-Tead-specific antibody. Tead-bound Malat1 was quantitated by qPCR. The nuclear RNA U6 was used as a negative control. **b.** Immunoblotting of Yap, Tead1, Tead2, Tead3,Tead4, and Hsp90 in B16F1, MEF, BMM, MSC, L929, and RAW264.7 cells. **c.** RNA pulldown assay. Unlabeled and biotinylated (Btn) Malat1 fragments (P1-P6) were synthesized by *in vitro* transcription (left panel), incubated with the lysate of HEK293T cells overexpressing V5-tagged Tead3, and pulled down with streptavidin beads. The bound proteins were eluted by boiling in Laemmli sample buffer and immunoblotted with a V5-specific antibody (right panel). **d.** Immunoblotting of Nfatc1, Ctsk, Tead1, Tead2, Tead3, Tead4, and β-actin in RAW264.7 cells treated with RANKL (50 ng/mL) for the indicated times. **e.** HEK293T cells co-transfected with SFB-Nfatc1 and MYC-Tead3 were subjected to pulldown with S-protein beads or a MYC-specific antibody, followed by immunoblotting with antibodies against MYC and FLAG. **f, g.** Control and MALAT1-knockout HEK293T cells co-transfected with SFB-Nfatc1 and MYC-Tead3 were subjected to pulldown with a MYC-specific antibody (**f**) or S-protein beads (**g**), followed by immunoblotting with antibodies against MYC and FLAG. **h, i.** MALAT1-knockout and Malat1-restored HEK293T cells co-transfected with SFB-Nfatc1 and MYC-Tead3 were subjected to pulldown with a MYC-specific antibody (**h**) or S-protein beads (**i**), followed by immunoblotting with antibodies against MYC and FLAG. **j.** Graphical representation of mouse Nfatc1 and Tead3 proteins and their truncation mutants. The upper panel shows full-length (FL) Nfatc1 and five truncation mutants: NT (N-terminal region), Mid (middle region = DNA-binding domain, DBD), CT (C-terminal region), NT + Mid, and Mid + CT. The lower panel shows full-length Tead3 and two truncation mutants: NT (N-terminal region = DNA-binding domain) and CT (C-terminal region = YAP-binding domain). **k.** HEK293T cells co-transfected with MYC-Tead3 and SFB-tagged full-length or truncated Nfatc1 were subjected to pulldown with S-protein beads, followed by immunoblotting with antibodies against MYC and FLAG. **l.** HEK293T cells co-transfected with SFB-Nfatc1 and MYC-tagged full-length or truncated Tead3 were subjected to pulldown with a MYC-specific antibody, followed by immunoblotting with antibodies against MYC and FLAG. **m, n.** HEK293T cells were co-transfected with Tead3, constitutively active Nfatc1 (Nfatc1-CA), a Renilla luciferase reporter, and a firefly luciferase reporter containing tandem Nfatc1-binding sites (**m**) or the *Ctsk* promoter (**n**). Luciferase activity was measured 48 hours after transfection. **o, p.** Wild-type, MALAT1-knockout, and Malat1-restored HEK293T cells were co-transfected with increasing amounts of Tead3, Nfatc1-CA, a Renilla luciferase reporter, and a firefly luciferase reporter containing tandem Nfatc1-binding sites (**o**) or the *Ctsk* promoter (**p**). Luciferase activity was measured 48 hours after transfection. **q, r.** Control and Malat1-knockdown RAW264.7 cells were transfected with Tead3 siRNA or scrambled negative control (NC). 24 hours after siRNA transfection, the cells were treated with RANKL for 5 days, followed by TRAP staining and quantification (**q**) of multinucleated TRAP-positive cells (outlined by dashed lines). Scale bars, 100 μm. Cell lysates were subjected to immunoblotting of Nfatc1, Ctsk, and β-actin (**r**). **s.** Model for the regulation of osteoclastogenesis by the Malat1–Tead3–Nfatc1 axis. Statistical significance in **a** and **m-q** was determined by a two-tailed unpaired **t**-test. Error bars are s.e.m.

To determine the role of Tead in Malat1-mediated regulation of Nfatc1, we first performed RNA immunoprecipitation assays, finding that Malat1 was enriched in pan-Tead immunoprecipitates from RAW264.7 cells (Fig. 4a), which validated the interaction between Malat1 and Tead in these osteoclast precursors. We next examined the protein levels of the four Tead family members (Tead1-4) in BMMs and RAW264.7 cells along with several other mouse cell lines. Interestingly, Tead1 and the Tead co-activator Yap were undetectable in BMMs and RAW264.7 cells but were abundantly expressed in the mouse melanoma cell line B16F1, mouse embryonic fibroblasts (MEF), mesenchymal stem cells (MSC), and the mouse fibroblast cell line L929 (Fig. 4b). In contrast, Tead3 showed a relatively specific expression pattern in BMMs and RAW264.7 cells (Fig. 4b). To determine whether Tead3 directly binds to Malat1, we performed RNA pulldown assays with six non-overlapping biotinylated fragments of Malat1 (P1-P6; 1.1-1.2 kb each) generated by *in vitro* transcription^22^, and we found that all six Malat1 fragments, but not an unrelated nuclear RNA U1, bound to Tead3 protein (Fig. 4c), suggesting that the Tead3-binding sites may be distributed diffusely on Malat1. Interestingly, RANKL treatment of RAW264.7 cells upregulated Tead3, but not other Tead family members (Fig. 4d). Further, co-immunoprecipitation (co-IP) assays revealed that Nfatc1 protein interacted with Tead3 protein (Fig. 4e) but not Yap protein (Extended Data Fig. 5b).

**Extended Data Figure 6.**
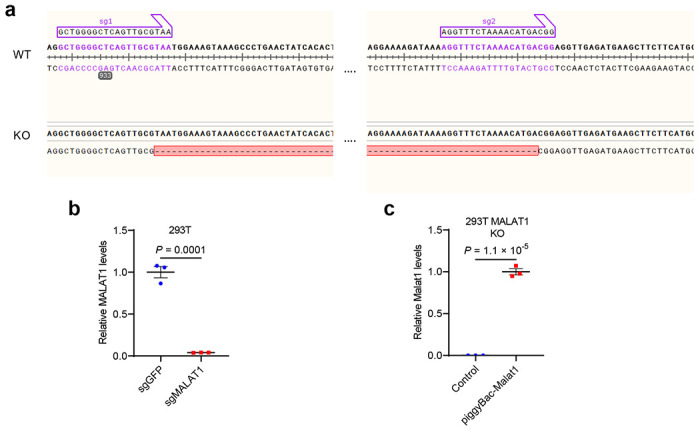
CRISPR-mediated MALAT1 knockout and transposon-mediated Malat1 re-expression. **a**. Schematic representation of the generation of MALAT1-null HEK293T cells by using a double excision CRISPR-knockout method. The MALAT1-knockout cells were verified by DNA sequencing as lacking the fragment between the two sgRNA sites. **b**. qPCR of MALAT1 in control and MALAT1-knockout HEK293T cells. **c**. qPCR of Malat1 in MALAT1-knockout HEK293T cells with or without piggyBac-mediated expression of Malat1.

After validating the interaction of Tead3 with Malat1 and Nfatc1, we sought to determine whether Malat1 modulates the binding of Tead3 to Nfatc1. To this end, we generated MALAT1-knockout HEK293T cells (Extended Data Fig. 6a, b) and transfected these cells with Tead3 and Nfatc1. Co-IP assays showed that Malat1 loss significantly increased the interaction between Tead3 and Nfatc1 (Fig. 4f, g). To further corroborate this result, we re-expressed Malat1 in MALAT1-knockout HEK293T cells (Extended Data Fig. 6c), finding that restoring Malat1 expression reduced the Tead3-Nfatc1 interaction (Fig. 4h, i). These results suggest that Malat1 may bind and sequester Tead3, thereby blocking Tead3 from associating with Nfatc1.

Nfatc1 protein contains four domains: two transcription activation domains (TAD) in N-terminal and C-terminal regions, a central DNA-binding domain (DBD), and an N-terminal regulatory domain (NHR)^47^ (Fig. 4j). Tead3 protein mainly consists of two domains: an N-terminal DBD (also known as the TEA domain) and a C-terminal YAP-binding domain^49^. Accordingly, we generated truncation mutants of Nfatc1 and Tead3 (Fig. 4j) and performed co-IP assays, finding that both the N-terminal region (containing a TAD and the NHR domain) and the central DBD, but not the C-terminal TAD of Nfact1, could bind Tead3 (Fig. 4k). In addition, co-IP assays using truncated Tead3 mutants and full-length Nfatc1demonstrated that the TEA domain of Tead3, but not the YAP-binding domain, was responsible for interaction with Nfatc1 (Fig. 4l).

**Extended Data Figure 7.**
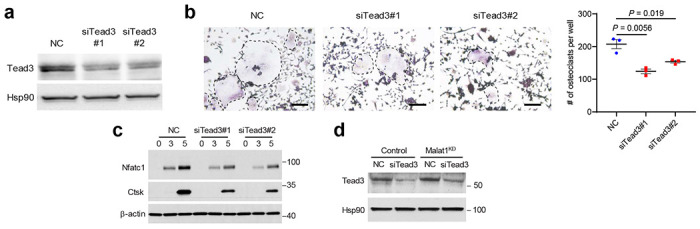
Effect of Tead3 knockdown on RANKL-induced osteoclastogenesis and expression of Nfatc1 and Ctsk. **a**. Immunoblotting of Tead3 and Hsp90 in RAW264.7 cells transfected with two independent Tead siRNAs or scrambled negative control (NC). **b**. TRAP staining images (left panel) and quantification (right panel) of control and Tead3-knockdown RAW264.7 cells treated with RANKL (50 ng/mL) for 5 days. Multinucleated TRAP-positive cells (outlined by dashed lines) were counted. Scale bars: 100 μm. Statistical significance was determined by a two-tailed unpaired *t*-test. Error bars are s.e.m. **c**. Immunoblotting of Nfatc1, Ctsk, and β-actin in control and Tead3-knockdown RAW264.7 cells treated with RANKL for 3 days and 5 days. **d**. Immunoblotting of Tead3 and Hsp90 in control and Malat1-knockdown RAW264.7 cells transfected with Tead3 siRNA or scrambled negative control (NC).

We further examined whether Malat1 and Tead3 modulate Nfatc1’s transcriptional activity by using a luciferase reporter containing either tandem Nfatc1-binding sites or the *Ctsk* promoter, and we found that overexpression of Tead3 indeed enhanced the transcriptional activity of Nfatc1 (Fig. 4m, n). We then transfected Tead3 into wild-type, MALAT1-knockout, and Malat1-restored HEK293T cells, finding that Tead3 overexpression led to higher dose-dependent increases in Nfatc1 activity in MALAT1-knockout cells compared with either wild-type or Malat1-rescued cells (Fig. 4o, p). These results lend support to a model in which Malat1 loss derepresses Tead3, which in turn binds and activates Nfatc1. Finally, to determine Tead3’s role in osteoclastogenesis, we knocked down Tead3 in RAW264.7 cells (Extended Data Fig. 7a), finding that Tead3 depletion attenuated RANKL-induced osteoclast differentiation (Extended Data Fig. 7b) and downregulated the expression of Nfatc1 and Ctsk (Extended Data Fig. 7c). Moreover, silencing Tead3 in Malat1-depleted RAW264.7 cells reversed RANKL-induced osteoclastogenesis (Fig. 4q, r and Extended Data Fig. 7d). Thus, Malat1 loss promotes osteoclast differentiation in a Tead3- and Nfatc1-dependent manner.

## Discussion

This study identified Malat1 as an osteoporosis-suppressing and bone metastasis-inhibiting lncRNA that is downregulated during RANKL-triggered osteoclastogenesis. RANKL stimulates multiple signaling pathways, most of which (such as MAPK and NF-κB pathways) can also be activated by other factors, and yet RANKL is indispensable and irreplaceable in osteoclastogenesis^14^, which could be explained by Nfatc1’s role as a specific master regulator of osteoclast differentiation. As a transcriptional factor of its own coding gene and other osteoclast-specific genes, the binding of Nfatc1 to other nuclear proteins can lead to synergistic activation of gene transcription^47^, as exemplified by the AP-1 transcription factor complex, which interacts with Nfatc1 to boost the transcriptional activity of Nfatc1^50^. Here, we identified Tead3 as a macrophage-osteoclast–specific Tead family member and a binding partner of Nfatc1, and our data suggest a model (Fig. 4s) in which Malat1 binds and sequesters Tead3, blocking Tead3 from associating with Nfatc1 and inducing the transcription of Nfatc1 target genes, including *Nfatc1* itself and *Ctsk*. In response to RANKL stimulation, downregulation of Malat1 releases Tead3, thereby enhancing both the Tead3-Nfatc1 interaction as well as the transcription factor occupancy of Nfatc1 target genes, which leads to activation of Nfatc1-mediated gene transcription and osteoclast differentiation.

In addition to hyperactivation of osteoclastic bone resorption, suppression of osteoblastic bone formation can also contribute to low BMD and osteoporosis. Several previous publications reported that MALAT1 promotes osteoblast differentiation by acting as a competing endogenous RNA (ceRNA) to microRNAs (miRNAs), i.e., a “miRNA sponge”, based on MALAT1 shRNA or siRNA knockdown in cell culture^51–55^. How a nuclear lncRNA could bind miRNAs is unclear. Considering the potential pitfalls in using RNAi, large genomic deletion (*MALAT1*, a single-exon gene, is ~7 kb in mice and ~8 kb in humans), promoter deletion, and RNase H-dependent antisense oligonucleotide approaches to deplete nuclear lncRNAs^15, 16, 56, 57, 58^, we used different methods for loss-of-function analyses of Malat1 *in vitro* and *in vivo*, including CRISPRi, double gRNA-mediated focal deletion in the 5′ region (without affecting the promoter), and insertional inactivation, along with genetic rescue experiments. In the present study, loss of Malat1 in pre-osteoclasts (including RAW264.7 cells and primary BMMs from *Malat1*^−/−^ mice) promoted osteoclastogenesis, a phenotype that could be reversed by restoration of Malat1 expression. On the other hand, the results from *Malat1*^+/+^, *Malat1*^−/−^, and *Malat1*^−/−^;*Malat1*^Tg/Tg^ mice, as well as osteoblast differentiation assays of MSCs isolated from these animals, showed no evidence for the regulation of osteoblastogenesis by Malat1 (Extended Data Fig. 3a–g).

Via its C-terminal YAP-binding domain, the TEAD family of transcription factors binds to the transcriptional co-activator YAP to turn on the expression of TEAD-YAP target genes^59^. In doing so, TEAD proteins and YAP are involved in several processes, including organ growth, regeneration, tumor progression, and metastasis^59^. Potentially druggable sites in the protein-protein interaction between YAP and TEAD, as well as a highly conserved palmitoylation pocket in TEADs, have been identified and exploited for drug development^60^. However, whether the TEAD family can function in a YAP-indepen dent manner is elusive. In this study, Tead3, but not other Tead family members, exhibited a specific expression pattern in macrophages/pre-osteoclasts, whereas Teadl, Yap, and classic Yap-Tead targets were barely detectable in these cells (Fig. 4b and data not shown). We further found that Tead3 binds and activates Nfatc1 via its N-terminal TEA domain (but not its C-terminal YAP-binding domain), which is required for RANKL-induced osteoclastogenesis, thus revealing a previously undescribed, non-canonical function of Tead that is mediated by Nfatc1 and is controlled by Malat1 lncRNA. Our findings suggest the therapeutic potential of developing agents that disrupt the TEAD3-NFATC1 interaction for treating osteoporosis and bone metastasis.

Future studies should address the following issues: first, we found that Malat1 expression is downregulated during osteoclast differentiation; yet, how this lncRNA is regulated by pro-osteoclastogenic factors under physiological and pathological conditions is unknown. Second, our study identified a Malat1–Tead3–Nfatc1 axis that regulates osteoclastogenesis, but it is possible that additional binding partners of Malat1 and Tead3 could also be involved in osteoclast differentiation. Third, our previous study^22^ and the present study collectively demonstrate that Malat1 binds to Tead to inactivate Yap-Tead’s pro-metastatic function in lung-metastatic breast cancer cells and to inhibit Nfatc1-Tead3’s pro-osteoclastogenic function in pre-osteoclasts, revealing both tumor-intrinsic and tumor-extrinsic mechanisms of Malat1 in metastasis suppression. Whether Malat1 suppresses metastasis at other anatomic sites (in addition to the lung and bone) and in cancer types in addition to breast cancer and melanoma warrants further investigation. Finally, whether Malat1 regulates other types of niche cells to control metastasis remains an open question.

## Methods

### Genetically engineered mouse models

All animal studies were performed in accordance with a protocol approved by the Institutional Animal Care and Use Committee of MD Anderson Cancer Center. Animals were housed at 70 °F-74 °F (set point: 72 °F) with 40%-55% humidity (set point: 45%). The light cycle of animal rooms is 12 hours of light and 12 hours of dark. Malat1-knockout mice with targeted disruption of *Malat1* (*Malat1*^−/−^) and mice with targeted transgenic expression of Malat1 from the *ROSA26* locus (*Malat1*^Tg/Tg^) were described in our previous paper^22^. To restore Malat1 expression in *Malat1*^−/−^ mice, we bred *Malat1*^−/−^ mice to *Malat1*^Tg/Tg^ mice and further mated their heterozygous offspring to produce *Malat1*^−/−^;*Malat1*^Tg/Tg^ mice. All mice described here were on a C57BL/6 background. Primers for PCR genotyping were listed in our previous paper^22^.

### Lipopolysaccharides (LPS)-induced inflammatory osteoporosis model

The procedure for inflammation-induced bone destruction was performed as previously described^36^. Briefly, 8-week-old female mice were injected above the calvarium with 12.5 mg/kg of LPS (Sigma, L4391) or vehicle (phosphate buffered saline, PBS). After 6 days, calvariae were collected and analyzed by micro-computed tomography (μCT), followed by embedding, sectioning, and TRAP staining.

### Micro-computed tomography (μCT)-based bone scanning and analysis

Mouse femurs were scanned on a Bruker micro-CT SkyScan 1276 (Bruker, Kontich, Belgium) with a source voltage of 55 kV, a source current of 200 μA, a filter setting of Al 0.2 mm, and pixel size of 13 μm at 2016 × 1344. We used 435 ms exposure time and the step and shoot mode with rotation step 0.400 degrees. Backward projection datasets of all femurs were reconstructed by using Insta-Recon software (Bruker microCT, Kontich, Belgium). Parameters for reconstruction were windowing 0-0.08 intensity, ring artifact reduction 5, beam hardening 23%, and automatic post-alignment correction. The proximal end of the femur corresponding to a 0-1.3 mm region (consisting of 100 sections over the region of interest) below the growth plate was selected and analyzed by using CTAn software (Version 1.18 8.0+, Bruker microCT, Belgium) to determine the trabecular bone mineral density (BMD), trabecular bone volume per tissue volume (BV/TV), trabecular number (Tb.N), and trabecular thickness (Tb.th). The threshold value for μCT was set at 150-250. All calculations were performed based on 3D standard microstructural analysis. For visualizing the femurs, a 3D model was created with CTVox software (Version 3.3.0, Bruker microCT, Belgium) based on the same region of the microstructural analysis.

Mouse calvariae were scanned on a Bruker micro-CT SkyScan 1276 (Bruker, Kontich, Belgium) with the same parameters as those used for scanning femurs, except the exposure time (400 ms), windowing (0-0.06 intensity), ring artifact reduction (3), and beam hardening (20). For visualizing the osteolytic area of the calvaria, a 3D model was created with CTVox software (Version 3.3.0, Bruker microCT, Belgium). A region of 8 mm × 8 mm centered at the midline suture was used for further quantitative analysis with ImageJ.

### Bone metastasis assay

B16F1 melanoma cells with stable expression of firefly luciferase (Addgene, 39196) were cultured to 70% confluence and harvested during the log phase of growth. Then, 5,000 cells were resuspended in sterile PBS, and the tumor cell suspensions were injected into the left tibiae of the 6-month-old *Malat1*^+/+^, *Malat1*^−/−^, or *Malat1*^−/−^;*Malat1*^Tg/Tg^ mice by using a 27-gauge needle under isoflurane anesthesia. Bioluminescence imaging (BLI) was performed at days 0, 14, and 25 after intratibial injection under isoflurane anesthesia by using an IVIS 200 imaging platform (Perkin Elmer), following the intraperitoneal injection of 100 μl D-luciferin substrate (25 mg/mL in PBS, Perkin Elmer). On day 26, the mice were euthanized. The tibiae were collected for *ex vivo* BLI imaging and photography. The imaging data were processed and quantitated with Living Image Software version 4.7 (Perkin Elmer).

### Histology, TRAP staining, and toluidine blue staining of bone tissues

The femurs and calvariae of mice were decalcified in 12.5% EDTA solution for 5 days before being transferred to 70% ethanol. After paraffin embedding, the tissues were sectioned at 4 μm thickness. The slides were deparaffinized in xylene, rehydrated in gradients of ethanol, and immersed in PBS for 5 min. For TRAP staining, we used a staining kit (Sigma, 387A-1KT) according to the manufacturer’s instructions. Briefly, the slices were incubated with the staining buffer containing Fast Garnet GBC solution, Naphthol AS-BI phosphoric acid solution, acetate solution, and tartrate solution in a 37 °C water bath protected from light for 1 hour. After being rinsed with water for 5 min, the slices were counterstained with methyl green (Vector Laboratories, H-3402-500) for 1 min and rinsed with water for 5 min. The slides were mounted with VectaMount Permanent Mounting Medium (Vector Laboratories, H-5000). For toluidine blue staining, the slides were stained with toluidine blue solution (Sigma, 89640), dehydrated and cleared with xylene, and coverslipped with DMX hydrophobic adhesive. All slides were scanned with an Aperio CS2 Digital Pathology Slide Scanner (Leica Biosystems). Bone histomorphometry was analyzed by using Bioquant OSTEO II software (Bioquant Nashville) on the sub-epiphyseal region 150 μm away from the distal growth plate and extending 1.3 mm into the bone compartment, at a distance of 150 μm from the cortical walls.

### Calcein staining of bone tissues

For dynamic histomorphometric measures of bone formation, calcein (Sigma, C0875) was intraperitoneally injected twice into mice (at 5 days and 1 day before euthanasia) at a dose of 25 mg/kg to obtain double labeling of newly formed bones. The non-decalcified femur bones were embedded in methyl methacrylate. The tissues were sectioned at 5 μm thickness, and the images were acquired by using an inverted microscope. Bone histomorphometric analysis of mineral apposition rate (MAR) was done with Bioquant OSTEO II software (Bioquant Nashville).

### Cell culture

The HEK293T cell line was from Li Ma’s lab stock (originally from the American Type Culture Collection, ATCC, CRL-3216). The L929 cell line was from Dr. Dihua Yu (MD Anderson Cancer Center, Houston, TX). The RAW264.7 cell line was from Dr. Liuqing Yang (MD Anderson Cancer Center, Houston, TX). The B16F1 cell line was from MD Anderson’s Cytogenetics and Cell Authentication Core. HEK293T, L929, RAW264.7, and B16F1 cell lines were cultured with Dulbecco’s Modified Eagle Medium (DMEM) supplemented with 10% fetal bovine serum (FBS) and 1% penicillin/streptomycin. Cells were maintained in a humidified, 5% CO2 atmosphere at 37 °C, and low-passage stocks were maintained in a centralized lab cell bank. Short tandem repeat profiling and mycoplasma tests were done by ATCC and MD Anderson’s Cytogenetics and Cell Authentication Core.

### Osteoclast differentiation

Osteoclast differentiation from bone marrow-derived macrophages (BMMs) was induced as described previously^61^. Briefly, femurs, tibiae, and iliac bones were removed from mice after euthanasia. Small incisions were made at both the proximal and distal ends of the bones, and the bones were placed in a sterile tube and centrifuged at 10,000 *g* at room temperature for 15 s. After purification with a 70 μm cell strainer, bone marrow cells were cultured in Minimum Essential Medium α (MEMα, Gibco, 41061029) containing 10% FBS for 1 day. Non-adherent cells were collected and seeded in 24- or 6-well plates and treated with 50 ng/mL of mouse M-CSF (Peprotech, 315-02) for 2 days, after which mouse soluble RANKL (Peprotech, 315-11) was added at a concentration of 100 ng/mL for additional culture for 4-6 days. Osteoclast differentiation from the RAW264.7 cell line was induced as described previously^62^. Cells were seeded in 24-well or 6-well plates at a density of 2 × 10^4^ or 5 × 10^5^ cells per well; 50 ng/mL of mouse soluble RANKL (Peprotech, 315-11) was used to induce differentiation, and the culture medium was changed every 2 days. Mature osteoclasts were identified as multinucleated (more than three nuclei) cells by TRAP staining (Sigma, 387A-1KT). Osteoclast markers (Nfatc1, Ctsk, and Trap5) were examined by qPCR and immunoblotting.

### Osteoblast differentiation

Osteoblast differentiation from bone marrow mesenchymal stem cells (MSCs) was induced as previously described^61^. Briefly, bone marrow cells were collected from the femurs, tibiae, and iliac bones of mice and plated for culture. After 48 h, non-adherent cells were removed, and attached cells were trypsinized and seeded in 12-well plates. When the cells reached 90% confluence, osteogenic differentiation medium (MEM containing 10% FBS, 5 mM β-glycerol phosphate, Selleck Chemicals, S3620, and 50 μg/ml of ascorbic acid, Selleck Chemicals, S3114) was added, and cells were cultured for 10-21 days. Then, alkaline phosphatase (ALP) staining was done by using a staining kit (Sigma, 86R-1KT) according to the manufacturer’s protocol. For ALP activity detection in the medium, equal volumes of conditioned medium were analyzed with an Alkaline Phosphatase Activity Fluorometric Assay Kit (BioVision, K422-500). For alizarin red S (ARS) staining, cells were fixed with 4% polyoxymethylene for 15 min and stained with 1% ARS (pH 4.2, Sigma-Aldrich, A5533) for 10 min. The dye was then removed and the cells were washed three times with water and photographed with an inverted microscope. The calcium mineralization stained with ARS was dissolved with 10% acetic acid and heated at 85 °C for 10 min, followed by neutralization with 10% ammonium hydroxide. The samples were transferred to 96-well plates and the absorbance at 405 nm was measured on a microplate reader (Biotek Synergy 2).

### F-actin ring staining

RAW264.7 cells were treated with RANKL to induce differentiation into osteoclasts, fixed with 4% paraformaldehyde for 20 min, rinsed with PBS, and permeabilized with 0.5% Triton X-100 at room temperature for 10 min. The cells were washed with PBS and blocked with 5% FBS at room temperature for 30 min. The fixed cells were stained with diluted phalloidin green 488 (1:100, BioLegend, 424201) in the dark for 20 min and mounted with the antifade mounting medium with DAPI (Vector Laboratories, H-1200-10). The slides were imaged with a Zeiss LSM880 confocal microscope and processed with Zen 2.6 (Zeiss) software.

### Plasmids

Mouse Nfatc1 was amplified from Prv-NFAT2 WT (Addgene, 11101) and Prv-NFAT2 CA (Addgene, 11102) by using PrimeSTAR Max DNA Polymerase (Takara, R045A). The resulting PCR products were subcloned into pDonor 201 through the Gateway BP reaction (Invitrogen, 11789020), and then cloned into the pBabe-SFB destination vector through the Gateway LR reaction (Invitrogen, 11-791-100). Mouse pDonor 223-Tead3 was obtained from the DNASU Plasmid Repository (https://dnasu.org/DNASU/Home.do) and cloned into pBabe-MYC and pLenti 6.2 FLAG-V5 vectors through the Gateway LR reaction (Invitrogen, 11-791-100). For cloning of truncation mutants, fragments of Nfatc1 or Tead3 were amplified and cloned into the pBabe-SFB or pBabe-MYC destination vector. Mouse Malat1 was amplified from pcDNA3.1-Malat1 (Li Ma’s lab stock) and subcloned into PB-CAG-BGHpA (Addgene, 92161) by using an In-Fusion Cloning Kit (Takara, 638909). Primers used for cloning are listed in **Supplementary Table 2**.

### Lentiviral transduction

Lentiviruses were produced in HEK293T cells by co-transfection with the viral vector and packaging plasmids (pMD2.G: Addgene, 12259; psPAX2: Addgene, 12260). Two days after transfection, viral supernatant was harvested, filtered through a 0.45 μm filter, and added to target cells in the presence of polybrene reagent (Sigma, R-1003-G) at 4 μg/mL. The infected cells were selected with puromycin, hygromycin B, or blasticidin as indicated below.

### Malat1 knockdown, knockout, and overexpression

To stably knock down mouse Malat1, we designed gRNAs against mouse *Malat1* by using CHOPCHOP (http://chopchop.cbu.uib.no/). The sgRNA sequences are listed in **Supplementary Table 3**. Primers were annealed and ligated into the pCRISPRia-v2 vector (Addgene, 84832) digested with BstXI and BlpI. RAW264.7 cells were infected with Lenti-dCas9-KRAB-blast (Addgene, 89567) lentivirus and selected with blasticidin (10 μg/mL). The surviving cells were then infected with pCRISPRia-v2-Malat1 lentivirus and selected with puromycin (10 μg/mL). Malat1 knockdown was verified by qPCR. To knock out MALAT1 in HEK293T cells, we infected the cells with lentiCas9-blast (Addgene, 52962) lentivirus and selected the cells with blasticidin (10 μg/mL). The surviving cells were infected with pDECKO GFP (Addgene, 72619) or pDECKO_Malat1_C (Addgene, 72622) lentivirus and selected with puromycin (1 μg/mL) as previously described^63^. After selection, single cells were plated in 96-well plates and cultured for 2 weeks. Malat1 knockout was verified by qPCR and DNA sequencing of individual clones. For restoration of Malat1 expression, MALAT1-knockout HEK293T cells were co-transfected with PB-CAG-BGHpA-Malat1 and Sleeping Beauty transposase (SB100: Addgene, 34879) by using polyethyleneimine hydrochloride MAX (Polysciences, 24765-1) and selected with hygromycin (300 μg/mL). qPCR was used to verify Malat1 re-expression.

### RNA interference

The siRNA Universal Negative Control (Sigma, SIC001) and siRNAs targeting mouse *Tead3* or *Nfatc1* were synthesized by Sigma. For mouse *Tead3*, siRNA constructs SASI-Mm02-00301288 (5′-CGUCUACAAGCUUGUCAAAdTdT-3′) and SASI-Mm02-00301289 (5′-GCAAGAUGUACGGUCGAAAdTdT-3′) were used. For mouse *Nfatc1*, siRNA constructs SASI-Mm02-00323571 (5′-CUCUCACGCUACAGCUGUUdTdT-3′) and SASI-Mm01-00029470 (5′-CCUCUGUGGCCCUCAAAGUdTdT-3′) were used. siRNA oligonucleotides were transfected into RAW264.7 cells by using Lipofectamine RNAiMAX (Invitrogen, 13778075) according to the manufacturer’s instructions. The knockdown efficiency was verified by immunoblotting.

### Cytoplasmic-nuclear fractionation

Control and Malat1-knockdown RAW264.7 cells were plated in 6-cm dishes. At 12 hours after seeding, the cells were treated with soluble RANKL (50 ng/mL) for 3 days. Nuclear and cytoplasmic proteins were fractionated by using the NE-PER Nuclear and Cytoplasmic Extraction Kit (ThermoFisher Scientific, 78833) according to the manufacturer’s protocol. After protein extraction, Western blot analysis was performed to detect Nfatc1 protein in the cytoplasmic and nuclear fractions. Gapdh and Lamin B1 were used as markers of the cytoplasm and the nucleus, respectively.

### Protein pulldown and immunoprecipitation

HEK293FT cells were transfected with SFB (a triple-epitope tag containing S-protein, FLAG, and streptavidin-binding peptide)-tagged Nfatc1 (full-length or truncation mutants) and MYC-tagged Tead3 (full-length or truncation mutants) and harvested 2 days after transfection. Cells were lysed in RIPA lysis buffer (Sigma, 20-188) at 4 °C for 15 min and sonicated. The lysates were centrifuged at 14,000 rpm at 4 °C for 15 min, and the supernatant was incubated with specific beads or antibodies. For pulldown of SFB-tagged proteins, cell extracts were incubated with S-protein beads (EMD Millipore, 69704-3). For immunoprecipitation of MYC-tagged proteins, cell extracts were incubated with anti-MYC beads (Sigma, A7470). After incubation at 4 °C overnight, the immune complexes were centrifuged and washed with PBS three times, and the bound proteins were eluted by boiling in Laemmli buffer at 95 °C for 10 min, followed by Western blot analysis with the indicated antibodies.

### Immunoblotting

Cells were lysed in RIPA lysis buffer (Sigma, 20-188, 10×: 0.5 M Tris-HCl, pH 7.4, 1.5 M NaCl, 2.5% deoxycholic acid, 10% NP-40, 10 mM EDTA) containing protease inhibitors and phosphatase inhibitors (GenDEPOT). Proteins were diluted in sample buffer (Bio-Rad), run on 4%-20% precast gradient gels (Bio-Rad), and transferred to a nitrocellulose membrane (Bio-Rad). After being blocked with 5% non-fat milk in Tris-buffered saline with 0.05% Tween-20, membranes were incubated with the primary antibody followed by the secondary antibody conjugated with horseradish peroxidase. After washing, the bands were detected with enhanced chemiluminescent reagent (ThermoFisher Scientific, 34580). Primary antibodies used are as follows: antibodies against Nfatc1 (1:1000, Santa Cruz Biotechnology, sc-7294), Tead1 (1:1000, Cell Signaling Technology, 12292S), Tead2 (1:1000, Proteintech, 21159-1-AP), Tead3 (1:1000, Proteintech, 13120-1-AP), Tead4 (1:1000, Proteintech, 12418-1-AP), Ctsk (1:1000, Proteintech, 11239-1-AP), Mitf (1:1000, Cell Signaling Technology, 12590S), Yap (1:1000, Cell Signaling Technology, 12395S), c-Fos (1:1000, Proteintech, 66590-1-Ig), c-Jun (1:1000, Cell Signaling Technology, 9165S), phospho-c-Jun (S63) (1:1000, Cell Signaling Technology, 91952S), p65 (1:1000, Cell Signaling Technology, 8242S), phospho-p65 (S536) (1:1000, Cell Signaling Technology, 3033S). Erk1/2 (1:1000, Cell Signaling Technology, 4695S), phospho-Erk1/2 (Thr202/Tyr204) (1:1000, Cell Signaling Technology, 4370S), Jnk (1:1000, Cell Signaling Technology, 9252S), phospho-Jnk (Thr183/Tyr185) (1:1000, Cell Signaling Technology, 4668S), IκBα (1:1000, Cell Signaling Technology, 9242S), Creb (1:1000, Cell Signaling Technology, 9197S), p38 (1:1000, Cell Signaling Technology, 8690S), FLAG tag (1:10000, Sigma, F7425 and F3165), MYC tag (1:2000, Cell Signaling Technology, 2278, and 1:2000, Santa Cruz Biotechnology, sc-40), HA tag (1:2000, Cell Signaling Technology, 3724S, and 1:5000, Santa Cruz Biotechnology, sc-7392), β-actin (1:4000, Santa Cruz Biotechnology, sc-47778), Gapdh (1:4000, Santa Cruz Biotechnology, sc-365062), and Lamin B1 (1:1000, Cell Signaling Technology, 13435S).

### RNA extraction, cDNA synthesis, and quantitative PCR (qPCR)

Total RNA from cells was extracted by using a TRIzol reagent (Invitrogen, 15596026) or a PureLink RNA Mini Kit (Invitrogen, 12183018A). cDNA was synthesized from 1 μg of total RNA by using an iScript cDNA Synthesis Kit (Bio-Rad, 1708891). Real-time PCR and data collection were done with SYBR Green Supermix (Bio-Rad, 1725124) on a CFX96 instrument (Bio-Rad). Data were normalized to *Actb*, *Gapdh*, or U6. The primer sequences are listed in **Supplementary Table 4**.

### Chromatin immunoprecipitation (ChIP) assay

ChIP assays of control and Malat1-knockdown RAW264.7 cells were done with a ChIP assay kit (Millipore, 17-371) as described previously^22^. Briefly, 1 × 10^7^ RAW264.7 cells were cross-linked by using 1% formaldehyde. Excess formaldehyde was quenched by glycine, and cell pellets were collected and lysed with SDS lysis buffer. The lysates were sonicated so that the chromosomal DNA fragments were 200-800 bp in length. Chromatin extracts were precleared with Protein G agarose, followed by immunoprecipitation with 8 μg of an Nfatc1-specific antibody (Invitrogen, MA3024) or normal mouse IgG at 4 °C overnight. Immune complexes were collected on Protein G agarose beads and washed. The protein-DNA complexes were eluted with 1% SDS in 50 mM NaHCO3. After the reversal of protein-DNA cross-links and removal of proteins, purified DNA was used for PCR-amplification of the *Ctsk* promoter region bound to Nfatc1. The *Ctsk* gene-specific primers were described previously^46^ and are listed in **Supplementary Table 5**.

### RNA pulldown assay

Full-length mouse Malatl (NR_002847) was divided into six non-overlapping pieces (PI-P6, 1.1-1.2 kb each) and cloned into the pGEM-T vector as previously described^22^. The vector was linearized by NotI-HF (New England Biolabs, R3189s) and used as the template for the synthesis of biotin-labeled RNA by using an *in vitro* T7 transcription kit (New England Biolabs, E2040S). Biotin-16-UTP (Roche, 11388908910) was used to biotinylate the RNAs. Non-biotinylated RNAs and biotinylated U1 were synthesized as negative controls. After *in vitro* transcription, the products were purified by using a PureLink RNA Mini Kit (Invitrogen, 12183018A) and digested with RNase-free DNase I (Invitrogen, 12185010) to remove the template DNA according to the manufacturer’s protocols. 3 μg of purified biotin-labeled or biotin-free RNA was heated at 90 °C for 2 min and chilled on ice for 2 min in the RNA structure buffer (2×: 20 mM Tris-HCl at PH 7.4, 0.2 M KCl, 20 mM MgCl_2_, 2 mM DTT) containing RNase inhibitor (Takara, 2313B). RNA samples were placed at room temperature for 20 min for proper secondary structure formation. HEK293T cells overexpressing V5-tagged Tead3 were lysed in RIPA lysis buffer (Sigma, 20-188) containing protease inhibitors (GenDEPOT) and RNase inhibitor and sonicated. Cell lysates were precleared with streptavidin agarose (Pierce, 20349) at room temperature. 3 mg of precleared cell lysates were added to each folded RNA sample and incubated at room temperature overnight. Streptavidin agarose was added and incubated at room temperature for 1 h. The bound proteins were washed and eluted by boiling in Laemmli buffer at 95 °C for 10 min and subjected to Western blot analysis.

### RNA immunoprecipitation (RIP) assay

The RIP assay was done with an EZ-Magna RIP Kit (Millipore, 17-10522) according to the manufacturer’s instructions. Briefly, approximately 4 × 10^7^ RAW264.7 cells were lysed in the Complete Nuclei Isolation Buffer (included in the kit) with protease inhibitor cocktail and RNase inhibitor and centrifuged at 800 *g* at 4 °C for 5 min. The nuclear pellet was resuspended with Complete RIP Lysis Buffer (included in the kit). After being treated with DNase I, the samples were precleared with Protein A/G magnetic beads and incubated with Protein A/G magnetic beads coated with a pan-Tead-specific antibody (Cell Signaling Technology, 13295S) or normal Rabbit IgG at 4 °C overnight. The beads were placed on a magnetic separator and washed with Nuclear RIP Wash Buffer, and the bead-bound immunoprecipitates were subjected to RNA purification by using a PureLink RNA Mini Kit (Invitrogen, 12183018A), followed by DNase I treatment. Purified RNA samples were used for cDNA synthesis, followed by qPCR analysis with the primers listed in **Supplementary Table 4**. U6 was used as a negative control. The results are presented as fold enrichment (normalized to IgG).

### Luciferase reporter assay

The pGL-NFAT reporter construct containing 3 × NFAT-binding sites was from Addgene (17870). The mouse *Ctsk* promoter region was PCR-amplified (PCR primers are listed in **Supplementary Table 2**) and ligated into the linearized pGL3-basic plasmid by using an In-Fusion HD Cloning Kit (Takara Bio, 638909). MALAT1 wild-type, MALAT1-knockout, and Malat1-restored HEK293T cells were plated in triplicates in 96-well plates. The next day, 16.67 ng of the indicated firefly luciferase vector, 50 ng of Nfatc1-CA-SFB, 1 ng of a Renilla luciferase vector, and 20 or 100 ng of Tead3-MYC were transfected per well. At 36 hours after transfection, firefly and Renilla luciferase activities were measured by using a Dual-Luciferase Reporter Assay (Promega, E1910) on a microplate reader according to the manufacturer’s protocol. Firefly luciferase activity was normalized to Renilla luciferase activity.

### ELISA

Mouse blood was collected through intracardiac puncture immediately after euthanasia. The blood was transferred to a 1.5 mL tube and left at room temperature for 30 min. The clot was removed by centrifuging at 7,000 rpm in a refrigerated centrifuge for 15 min. The resulting supernatant was aliquoted and stored in a −80 °C freezer. For the detection of serum TRAP5b, a TRAP5b ELISA Kit (Immunodiagnostic Systems, SB-TR103) was used according to the manufacturer’s instructions.

### Bioinformatic analysis

The RNA-seq data of hematopoietic stem cells (HSCs) and different hematopoietic lineages from umbilical cord blood of healthy individuals (E-MTAB-3819) or mouse bone marrow (E-MTAB-7391) were downloaded from Expression Atlas (https://www.ebi.ac.uk/gxa/experiments). A heatmap of differentially expressed genes was generated by using R language. Human MALAT1 expression was compared among HSCs, multipotent progenitors (MPPs), and common myeloid progenitors (CMPs). The expression value in each group is the average of three or four healthy individuals. Mouse Malat1 expression was compared among HSCs and four hematopoietic multipotent progenitors (MPPs): MPP1, lineage-Sca1+Kit+CD135+CD150−CD48+CD34+; MPP2, lineage-Sca1 +Kit+CD135−CD150+CD48+CD34+; MPP3, lineage-Sca1+Kit+CD135−CD150+CD48−CD34+; and MPP4, lineage-Sca1+Kit+CD135−CD150−CD48+CD34+. The expression value in each group is the average of four samples, and each sample contains cells pooled from three mice.

The RNA-seq data (GSE38747) of CD14+ macrophages and the derived multinucleated giant cells (MGCs) were downloaded from Gene Expression Omnibus (GEO; (http://www.ncbi.nlm.nih.gov/geo/). After the probe ID was converted to the gene symbol based on the annotation of the platform, the data were normalized and an expression matrix was obtained. The differentially expressed genes (DEGs) were identified by limma package in R language with the following cut-off values: |log_2_ (fold change)| > 1 and *P* value < 0.001. The heatmap and volcano map of the DEGs were created by R language, and the DEGs were subjected to Gene Set Enrichment Analysis (GSEA) through clusterProfiler and enrichplot package in R^64^. Gene sets with an adjusted *P* value of less than 0.05 were considered significantly enriched and are listed in **Supplementary Table 1**.

### Statistical analysis

Except for the animal experiments, all experiments were repeated two to three times. Statistical analyses were done with GraphPad Prism 9.0. Unless otherwise noted, data are presented as mean ± s.e.m., and a two-tailed unpaired *t*-test was used to compare two groups of independent samples. Statistical methods used for RNA-seq analysis and Expression Atlas data analysis are described above. *P* < 0.05 was considered statistically significant.

## Extended Data
